# Circulating miRNAs are Down-regulated in Asthmatic Patients; Case-control Study

**DOI:** 10.7150/ijms.111022

**Published:** 2025-09-08

**Authors:** Christian A. Trejo-Jasso, Héctor Isaac Rocha-González, Eduardo Montes-Martínez, Manuel Castillejos-López, Arnoldo Aquino-Galvez, Dora Patricia Rosete-Olvera, Jose S. Lopez-Gonzalez, Mario Perez-Medina, Martha Patricia Sierra-Vargas, Angeles Carlos-Reyes, Misael O. García-Martin, Jazmín García-Machorro, Angel Camarena, Victor Ruiz

**Affiliations:** 1 Escuela Superior de Medicina, Departamento de Posgrado, Instituto Politécnico Nacional, Mexico City 11340, Mexico.; 2 Laboratorio de Biología Molecular, Departamento de Fibrosis Pulmonar, Instituto Nacional de Enfermedades Respiratorias Ismael Cosío Villegas, Mexico City 14080, Mexico.; 3 Departamento de Epidemiología Hospitalaria e Infectología, Instituto Nacional de Enfermedades Respiratorias Ismael Cosío Villegas, Mexico City 14080, Mexico.; 4 Departamento de Virología, Instituto Nacional de Enfermedades Respiratorias, Ismael Cosío Villegas, Mexico City 14080, Mexico.; 5 Laboratorio de Cáncer Pulmonar, Departamento de Enfermedades Crónico-Degenerativas, Instituto Nacional de Enfermedades Respiratorias Ismael Cosío Villegas, Mexico City 14080, Mexico.; 6 Departamento de Investigación en Toxicología y Medicina Ambiental, Instituto Nacional de Enfermedades Respiratorias Ismael Cosío Villegas, Mexico City 14080, Mexico.; 7 Laboratorio de Onco-Inmunobiologia, Departamento de Enfermedades Crónico-Degenerativas, Instituto Nacional de Enfermedades Respiratorias, Mexico, Mexico.; 8 Laboratorio de Medicina de Conservación, Escuela Superior de Medicina, Instituto Politécnico Nacional, Mexico City 11340, Mexico.; 9 Laboratorio de Inmunobiología y Genética, Instituto Nacional de Enfermedades Respiratorias Ismael Cosío Villegas, Mexico City 14080, Mexico.

**Keywords:** Asthma, biomarkers, micro-ribonucleic acids, miR-17-5p, miR-106b-3p, miR-126-3p, miR-223-3p

## Abstract

**Purpose:** Microribonucleic acids (miRNAs) play an important role as non-invasive biomarkers and predictors of inflammatory disease activity. This study aimed to determine differences in expression profiles of miRNAs in patients with asthma compared to healthy controls (HC).

**Study Design and Methods:** In this study, we evaluated a panel of 84 miRNAs in plasma from 3 asthmatic patients and three healthy controls (HC), and we observed decreased levels of miR-17-5p, miR-18a-5p, miR-106b-5p, miR-126-3p, miR-223-3p y miR-374a-5p which were validated in an independent group. Real-time PCR was performed to compare the expression level of each miRNA in a group of 27 asthmatic patients and 27 healthy controls.

**Results:** Correlations between miRNAs and body mass index (BMI) and forced expiratory volume during the first second (FEV1) were also determined. Asthmatic patients had lower levels of miR-17-5p(p=0.026), miR-106-5p (p=0.022), miR-126-3p (p=0.041, and miR-223-3p (p=0.13) than healthy controls. miRNAs did not correlate with BMI and FEV₁.

**Conclusion:** Data suggest that circulating miRNAs described in this study could be employed as biomarkers for asthma prediction.

## Introduction

Asthma is a respiratory disease that affects individuals of all ages. It is now recognized as a condition with several phenotypes and a group of diseases known as endotypes. Some asthma phenotypes that have been described include young individuals with allergies, overweight middle-aged individuals, and elderly individuals with unhealthy aging, among others. However, their similarities give rise to a common syndrome characterized by reversible airway obstruction, nonspecific airway hyperresponsiveness, and chronic airway inflammation 1, 2. According to the Global Burden of Diseases, Injuries, and Risk Factors Study (GBD), the number of prevalent cases and deaths of asthma continues to increase, with an estimated 262.41 million prevalent cases globally 3. Its prevalence and severity in Latin America are high 4. In Mexico, asthma prevalence is 5% and 6.9%, respectively, in the adult and childhood populations 5,6. Asthmatic patients often present exacerbations of symptoms, which increase the level of bronchial inflammation and hyperresponsiveness. Both processes induce the remodeling of the airways, with a decrease in lung function 7,8. The underlying pathogenesis of asthma is highly complex and diverse, with a significant economic impact due to the need for long-term treatment 9 and a potential decrease in quality of life. Clinically, asthma is a chronic airway disease characterized by recurrent episodes of wheezing, coughing, thoracic oppression, and dyspnea 10. The immune system plays a central role in the pathophysiology of asthma, involving the inflammatory response and sensitivity to allergens 11. In this context, various biomarkers have been studied to monitor the evolution and prognostic of patients with asthma 8,12.

Micro-ribonucleic acids (miRNAs) are short, non-coding ribonucleic acids of about 18-24 nucleotides long, involved in silencing gene expression by degradation or translational repression. In addition, miRNAs regulate the gene expression of a wide variety of code genes to proteins. Therefore, its deregulation could lead to human disease. miRNAs modulate allergic immune responses, being good candidates for developing novel therapies and as biomarkers for the diagnosis, activity, and prognosis 13. As biomarkers, miRNAs can be quantified by cost-effective, noninvasive methods because they can be isolated from serum/plasma or other biological fluids such as nasal mucosa and saliva 14. In asthma, some miRNAs have been identified to increase or decrease their levels. Also, they are related to the inflammatory response 15. The importance of miRNAs in asthma has also been described in the allergic reaction of the disease, where a specific miRNA profile has been identified 16. The study of miRNAs in asthma has allowed the identification of miRNA genes, which are considered biomarkers due to their importance in the pathological process 17. In this context, miRNA biomolecules play a great role in the genetic regulation of various genes, mainly those related to the pathophysiology of the disease. Therefore, it is fundamental to focus on possible prognostic predictors that could identify patients with a high risk of developing severe forms of the disease. This study aimed to determine differences in expression profiles of miRNAs in patients with asthma compared to healthy controls (HC).

## Study Design and Methods

### Population studied

Fifty-eight patients over 18 years of age, both genders, with a clinical diagnosis of mild asthma with at least six months of treatment, and who had presented the following symptoms: soft wheezing, cough, and minor difficulty breathing were included. They were selected from the "Asthma Clinic" of the Instituto Nacional de Enfermedades Respiratorias “Ismael Cosío Villegas” of Mexico City. The diagnosis of asthma and the degree of severity were based on the GINA guidelines. Patients with congenital malformation of the airways or other chronic lung diseases such as Chronic Obstructive Pulmonary Disease (COPD), pulmonary fibrosis, bronchi-ectasis, allergic bronchopulmonary aspergillosis, and lung cancer were excluded from the study. Laboratory parameters, such as serum total IgE levels and cell count were evaluated in asthmatic patients. Atopy was identified by measuring the level of specific immunoglobulin E and eosinophil count in serum and confirmed by the clinical patient´s history, taking these parameters into account, we can consider our group of asthmatic patients to have a T2 high endotype according to the GINA and GEMA guidelines. The control group fulfilled similar characteristics (age and sex). Thirty-three individuals were clinically healthy, unrelated subjects, had no family history of asthma, allergies, or atopy, with no clinical evidence of respiratory diseases such as COPD, IPF, bronchopulmonary aspergillosis, or lung cancer, and with no clinical history of diabetes, degenerative autoimmune, or chronic illnesses. A questionnaire, medical history, and clinical examination determined the clinical status of Healthy Controls (HC). Smokers were not included in any group. Data demographics are shown in Table 1. The Institutional Research and Bioethics Committee of the National Institute of Respiratory Diseases reviewed and approved the protocols for genetic studies under which all subjects were recruited. All patients who participated in the study signed informed consent forms.

Plasma from subjects was obtained cross-sectionally using a blood collection tube containing EDTA and stored at -70 °C. The miRNA was obtained from 200 µL plasma samples from total RNA using the miRNeasy Serum/Plasma kit (Qiagen, Germany) with the addition of the C. elegans miR-39 (cel-miR-39) miRNA mimic (Qiagen, Germany) as a spike-in control according to the manufacturer's protocol. Total RNA was reverse transcribed into cDNA using the miScript II RT kit miScript HiSpec buffer (Qiagen, Germany).

### miRNAs expression levels

**Figure 1:**
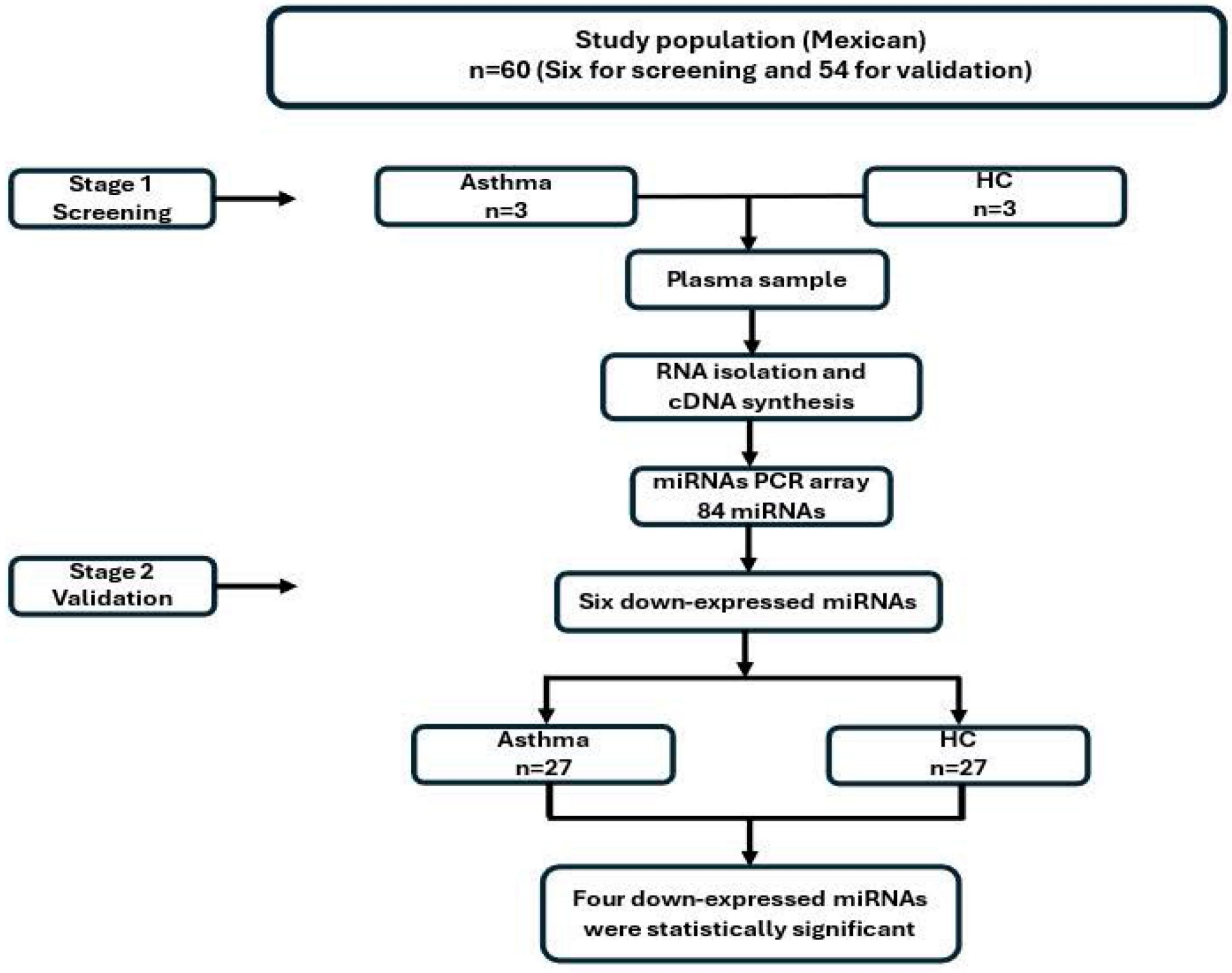
** Methodological strategy**.

Our study was developed in two stages. First, we analyzed three asthmatic patients and three HC. In the second stage, we evaluated fifty-five patients with asthma (thirty-seven women, and eighteen men) and thirty healthy individuals (twenty-three women, and seven men) as a control group (Figure 1).

### First stage: screening of miRNA expression

Three subjects were randomly selected from both groups. The expression was evaluated using a pre-designed miScript miRNA PCR Array (Qiagen, Germany). This kit can detect the expression of 84 miRNAs in plasma. This stage aimed to detect more than 2-fold differences between asthmatic and HC. miRNA quantitation was performed following manufacture instructions; briefly, a PCR master mix was made of miRScript universal primer, free RNAse water, and 0.5ng of cDNA. The qPCR array was performed using the miScript SYBR Green PCR kit (Qiagen, Germany) following the manufacturer´s instructions with the Quant Studio 12k flex PCR. The results were analyzed on the site: http://pcrdataanalysis.sabiosciences.com/mirna/arrayanalysis.php. However, this page is discontinued. The distribution of each population was compared with the Student-t test, with an α value of 0.01, statistical error of 10%, and 99% of power. The expression of six miRNAs was decreased more than 2-fold in the asthmatic group (miR-17-5p, miR-18a-5p, miR-106b-5p, miR-126-3p, miR-223-3p, and miR-374a-5p).

### Second stage: validation of miRNAs down-regulated

This stage used independent samples of patients with the same characteristics as those in the first stage. The expression of 6 miRNAs was analyzed employing the real-time RT-PCR method. Briefly, 10 ng of total RNA was converted into cDNA using kit TaqMan microRNA reverse transcription (ThermoFisher Scientific, USA) following manufacturer instructions. After that, 10 ng of cDNA was quantified by real-time PCR using a specific TaqMan probe for each miRNA using FG Taqman GEx Master Mix 2X (Termofisher Scientifics, USA). Cycle conditions were one step of 10 min 95 °C, 40 cycles of 95 °C, 15s, and 60 °C, 60s. Relative plasma miRNA levels were normalized to miR-30e-5p expression levels in asthmatic and healthy subjects to calculate 2-Δct (Ct miRNA- Ct miR-30e-5p). The qPCR was carried out following the manufacturer´s instructions with the Quant Studio 12k flex PCR.

### Statistical analysis

Comparisons of the distribution of demographic data and clinical characteristics were performed with the T-student test, and 2-ΔΔct miRNA levels were performed with the Mann-Whitney U test. The Pearson test was used to analyze the correlation of distributions. The comparison of frequencies was determined with χ2. In the Kyoto Encyclopedia of Genes and Genomes (KEGG) (http://62.217.122.229:3838/app/miRPathv4), we analyzed the pathway using DIANA-mirPath v4. The miRNA target genes were predicted using TarBase v8.0 genes intersections with a prediction threshold 0.5. Significantly enriched KEGG pathways were selected with Fischer Exact test.

## Results

### Demographic and clinical characteristics

Table 1 shows the demographic and clinical characteristics of the asthmatic population and control group. There were no differences in sex; however, significant differences were observed when analyzing age, weight, and body mass index (BMI). The asthmatic group was overweight compared with the HC. Finally, forced expiratory volume during the first second (FEV1) showed differences between both groups because of the asthma condition. There was no correlation between miRNA and demographic covariates, such as age, weight, BMI, and FEV1. According to the GINA and GEMA guidelines 18-19. IgE levels, which were above the reference value of 150 IgE U/ml in 30% of the patients analyzed, indicate that in our group of asthmatic patients, 30% of them are atopic. Another important data point is the percentage of eosinophils in our group of asthmatic patients, which was above 3% in 78.4% of the asthmatic patients, and the eosinophil count was above 150 in 83.1% of the asthmatic patients. Complete data in Table S1. Therefore, we consider our study group to have eosinophilic asthma, that is, a T2 High asthma endotype (Table 2).

### Relative quantification of miRNAs by microarrays (First stage)

The most common method for relative quantitation is the 2^-ΔΔCt^ method, which calculates the relative fold gene expression when performing real-time PCR 20. In the first stage, we were able to identify differences in the expression of six miRNAs downregulated, miR-17-5p, miR-18a-5p, miR-106b-5p, miR-126-3p, miR-223-3p, and miR-374a-5p. The expression was evaluated according to the manufacturer's protocol. We observed statistical significance when comparing differences in the expression levels Log_2_(2^-ΔΔct^) of miRNAs in the group of patients regarding HC. Data are shown in Table 3.

Besides, when we stratified by gender and BMI, these variables do not modify the expression of 2^-ΔΔct^ miRNA levels (Data not shown). However, in asthmatic patients, down expression of miR-17-5p, miR-18a-5p, miR-106b-5p, miR-126-3p, miR-223-3p, and miR-374a-59, was observed. In previous studies, these miRNAs have been associated with prognostic and/or diagnostic biomarkers in asthma.

### Validation of miRNAs down-regulated (Second Stage)

**Figure 2:**
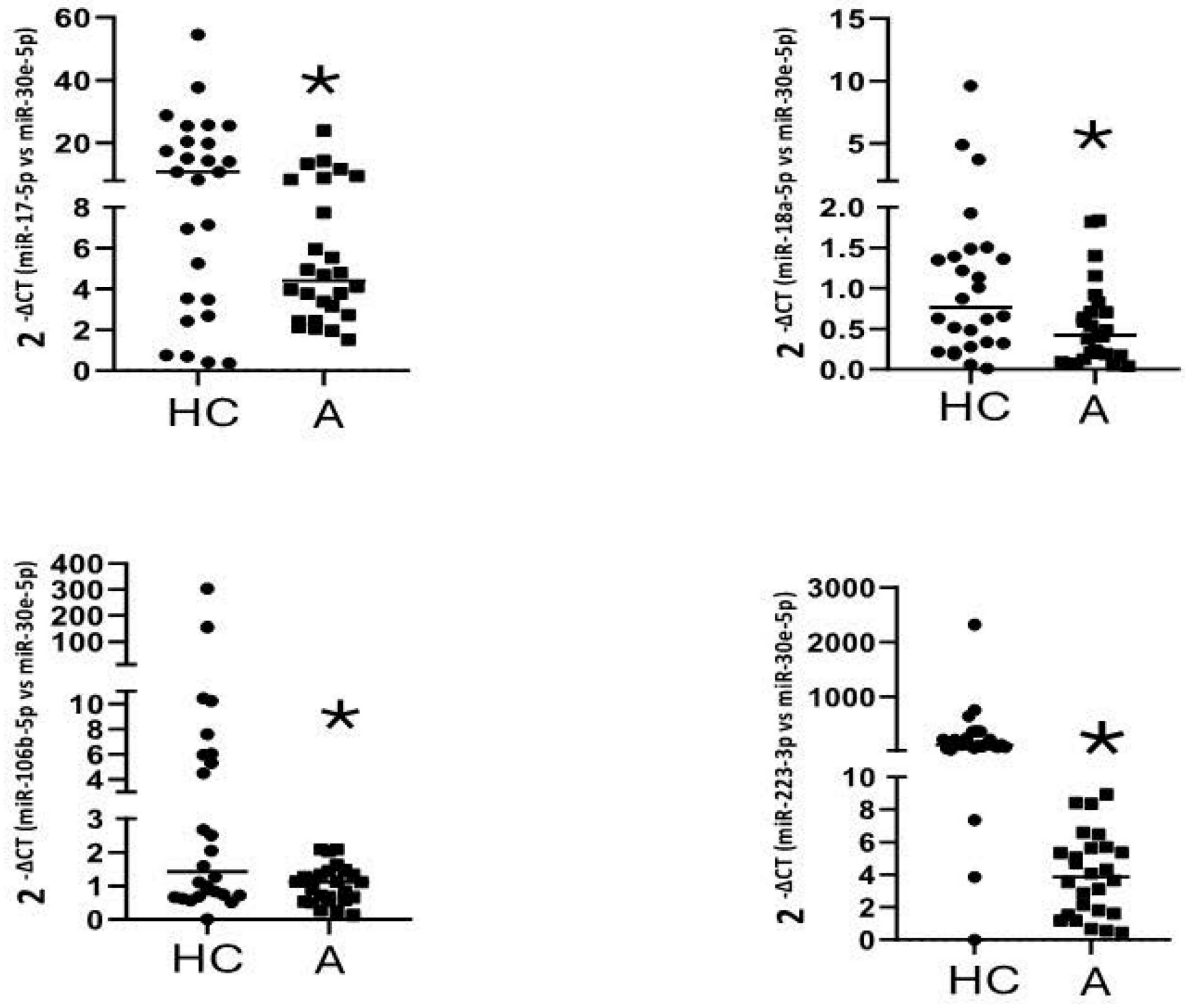
Down-regulation of gene expression of miR-17-5p (*p=0,026), miR-106b-5p (*p=0,022), miR-18a-5p (*p=0,041), and miR-223-3p (*p=0,013) in plasma samples of asthmatic subjects. Data are presented as the median and interquartile range of 2^-Δct^. *Significantly different from HC by Mann-Whitney U test with a p < 0.05. A=Asthmatic group. HC=Healthy control.

The six miRNAs found downregulated in the first stage were evaluated in fifty-five asthmatics and thirty healthy controls. In the validation stage, there was no correlation between the expression levels of miRNAs and the group of patients studied, however, when matching by age, comparing 27 healthy subjects (46.66 ± 12.53, age) and 27 controls (45.44 ± 12.48, age), we found differences between the asthmatic group versus healthy controls for miR-17-5p, miR-18a-5p, miR-106b-5p, and miR-223-3p (Figure 2) (Table 4).

### Kyoto Encyclopedia of Genes and Genomes analysis

**Figure 3:**
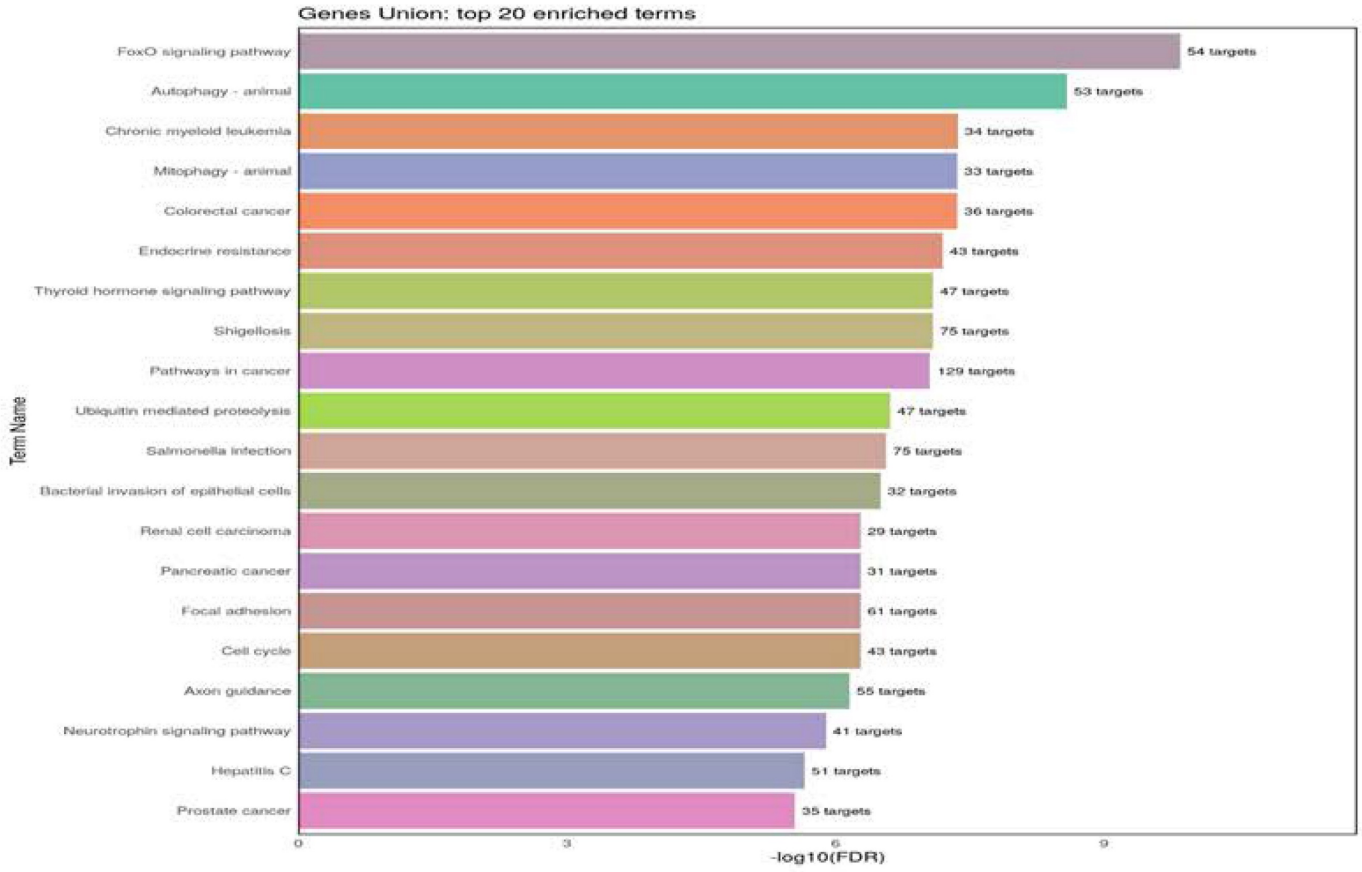
Kyoto Encyclopedia of Genes and Genomes (KEGG) pathways analysis of four miRNAs as asthma-HC. The figure indicates the number of pathways union of four miRNAs. The gen list is significantly enriched KEGG pathways (p <0.02) are listed in rows and ranked based on the number of a target gene*. (Targets resource: TarBase v8.0, | Terms/Pathways resource: KEGG | Species: Homo sapiens)*

For the Kyoto Encyclopedia of Genes and Genomes (KEGG) analysis, we selected those pathways shared by miR-17-5p, miR-18a-5p, miR-106b-5p, and miR-223-3p. The analysis indicates the twenty KEGG biological pathways based on the number of target genes associated with these miRNAs. The more significant pathways are FoxO and autophagy, which involve four miRNAs (Figure 3). The messenger RNA targets of the FoxO pathway are *Akt1, FoxO1, FoxO3, STAT3, TGFβ r1, and TGFβ r2* (supplementary material). Examples related to messenger RNA autophagy targets are *Akt1, 2, and 3, ATG16L1, ATG2B, and ATG3.* (supplementary material, Table S2).

## Discussion

Asthma is one of the respiratory diseases with the most significant incidence and socioeconomic burden worldwide. It is a complex disease, and it is the result of the interaction between genetic and environmental factors and lifestyle. Thus, novel markers, such as circulating miRNAs, could offer new diagnostic, therapeutic, and prognostic alternatives. miRNA molecules have been widely studied in chronic and infectious diseases because they modulate various molecules and cellular receptors that regulate the immune response.

Some studies have found an association between asthma and miRNA expression in different biological samples. Several miRNAs have been identified as biomarkers and related to lung function decline. In addition, asthmatic patients show heterogeneity in their treatment response, and some biomolecules such as miRNAs have been identified as predictive biomarkers of response to pharmacological therapies; however, the results have been contradictory.

In this study, we identified six miRNAs down-regulated in the group of asthmatic patients in the first stage of analysis, and only validated four miRNAs in the second stage. Regarding miR-17-5p, miR-18a-5p, miR-106b-5p, and miR-223-3p, which have been proposed as biomarkers in asthma, some studies have shown that miR-17-5p, miR-18a-5p, and miR-106-3p, which belong to the miR-17 ~ 92 cluster family, interact with the same messenger RNAs and due to their relationship with asthma have been previously proposed as diagnostic biomarkers 14, 21.

In this regard, miR-17-5p suppressed the proinflammatory effect in a model of mice with ALI. This result is because toll-like receptor 4 (TLR-4) and nuclear factor (Nf)-kB inflammatory signaling pathway, was targeted by miR-17-5p 26. Other authors have demonstrated that the stimulation of TLR-4/(Nf)-kB is involved in the development of allergic asthma in various murine models. 27 Which corroborates our findings of a decrease in miR-17-5p. In the same way, stimulation of macrophages by lipopolysaccharides induces a reduction of mir-223 and an increase of STAT-3 levels, a proinflammatory cytokine 28. The suppression of (Nf)-kB and STAT-3 through I cariside II (a flavonoid) inhibits the development of eosinophil-mediated airway inflammation 29. Similarly, recombinant IL-37 upregulated IκB expression and downregulated levels of NF-κB p65, phospho-NF-κB p65, STAT3 and phospho-STAT3 both in OVA-induced mice 30 Although these effects have been observed in murine asthma models, the expression of miR-17-5p appears to induce the same effect as Icariside II and recombinant IL-37. A study conducted by Chen et al. in asthmatic patients demonstrated that plasma levels of miR-17-5p are decreased. Using bioinformatic tools such as TargetScan, the authors predicted that miR-17-5p may negatively regulate the LIGHT gene (TNFSF14), a cytokine involved in airway inflammation and remodeling. 31 Previous studies have demonstrated that decreased levels of miR-17-5p are associated with increased inflammatory processes in asthma.

The decreased expression of miR-18a-5p is associated with increased *in vitro* differentiation of CCR6⁺ and RORγt⁺ Th17 cells (RAR-related orphan receptor γt). Moreover, it is also linked to an increase in the number of tissues Th17 cells expressing CCR6, RORγt, and IL-17A in *in vivo* airway inflammation models. This relationship was demonstrated in the study, where it was observed that miR-18a deficiency in CD4⁺ T cells lead to enhanced differentiation toward the Th17 phenotype. Additionally, in murine models of airway inflammation, a significant increase in Th17 cells was found in the lungs of miR-18a-deficient mice, as evidenced by elevated expression of CCR6, RORγt, and IL-17A. This study provides strong evidence for the inhibitory role of miR-18a in Th17 cell differentiation and its involvement in airway inflammation. 32 The differentiation of Th17 cells is regulated by several cytokines, including the combination of IL-6 and TGF-β1. It has been demonstrated that Th17 phenotype lymphocytes actively participate in the inflammatory process observed in asthma. 33

Moreover, a study conducted on bronchial epithelial cells derived from asthmatic patients showed that the levels of both miR-106b-5p and miR-18a-5p were decreased compared to those in bronchial cells from healthy individuals. To investigate the role of these miRNAs, the authors performed an in-silico analysis using the mirPath software (DIANA-microT_v4.0). This analysis indicated that these miRNAs may regulate inflammatory signaling as well as the TGF-β, IFN, and IL-6/IL-8 pathways 34

This finding is corroborated by our study in asthmatic patients, who exhibit reduced plasma levels of miR-18a-5p compared to healthy individuals.

Through downregulating of TRAF6 expression in cardiac macrophages, miR-223 can regulate the inflammatory process 35. On the other hand, exosomes, are nanoscaled vesicles (30-100 nm in diameter) produced by various cells, and contain enriched amounts of biomolecules 36. Exosomes are among the most important modulators of inflammatory diseases found in different organs such as the lung (lung cancer, COPD, asthma). One of their main functions is to act as transporters of a wide range of molecules, such as proteins, lipids, and microRNAs (miRNAs). Therefore, exosomes could influence several physiological and pathological processes, including those involved in asthma. They can be detected in multiple cell types and biofluids, providing a wealth of information about the processes in a pathological scenario 37. He et al, found that exosomes derivate of healthy mice bronchoalveolar lavage loaded with miR-223-3p mimics/inhibitors alleviates acute lung injury (ALI) by targeting STK39 in alveolar macrophages 38. These studies showed a significant association of mir-223 in the negative regulation of inflammatory cytokines in macrophages. However, in two independent cohorts, expression of miR-629-3p, miR-223-3p, and miR-142-3p was significantly upregulated in the sputum of patients with severe asthma compared with that in healthy control subjects and the levels of these miRNAs were highest in patients with neutrophilic asthma 39.

In an experimental model of asthma, overexpression of miR-223 and miR-21 improves the conversion of stem cells to eosinophils. The action of miR-223 is through IGF1R present in eosinophils, it was proposed for this research 40. The overexpression of miR-223 decreased the production of extracellular matrix proteins, α-SMA, and type I and II collagen. Furthermore, inhibition of it was observed to induce opposite effects in airway smooth muscle cells by the PI3K / Akt pathway and the insulin-like growth research receptor type 1 41. In a murine model of asthma, the authors demonstrated that miR-223-3p plays a protective role in neutrophilic asthma by inhibiting the NLRP3 inflammasome, a multiprotein complex that assembles in immune cells and triggers inflammatory processes 42. In line with this experimental study, our results from the plasma of asthmatic patients further support that the downregulation of miR-223-3p may be associated with the development of asthma.

The KEGG analysis shows different biological signaling pathways, with FOXO and autophagy pathways as major pathways. Transcription factor FoxO-1 is inhibited by Tranilast, a drug used for bronchial asthma treatment 43. Likewise, FoxO1 can regulate allergic asthmatic inflammation through the polarization of macrophages toward a pro-inflammatory phenotype. In response to allergenic challenges, FoxO1 overexpression in macrophages has been associated with lung inflammation in asthma 44 In the same way, proteins described in the pathway of FOXO, such as transforming growth factor-beta receptor type 1 (TGFβ-R1) and receptor type 2 (TGFβ-R2) are associated with the airway remodelation process 45. Given that the FoxO1 pathway is regulated by the four miRNAs whose expression is downregulated in our asthmatic patients, it can be inferred that FoxO1 may be actively involved in the inflammatory process observed in these patients.

Regarding autophagy, a study conducted in three independent cohorts of children with asthma identified two promoter polymorphisms in the ATG5 gene (rs12201458 and rs510432)—which is essential for autophagosome formation—as being associated with pediatric asthma 46. Another study compared autophagy levels in granulocytes derived from sputum and peripheral blood cells (PBCs) among patients with severe asthma, non-severe asthma, and healthy subjects. The results demonstrated that individuals with severe asthma exhibited significantly higher levels of autophagy compared to those with non-severe asthma and healthy controls 47. Given that autophagy pathways, including ATG5, may be regulated by miRNAs that are downregulated in our asthmatic patients, these findings further support the notion that altered miRNA expression may contribute to asthma pathogenesis by modulating key processes such as autophagy 47. A study conducted in Atg5 knockout mice demonstrated reduced inflammation and decreased airway hyperresponsiveness in an ovalbumin-induced asthma model 48. On the other hand, Wnt5a-Ca2+/CaMKII-autophagy axis can induce airway remodelation 49.

Apparently, this autophagy mechanism in airway cells, whether in *in vitro* or *in vivo* models, is significantly associated with the inflammatory process present in asthma, and this process is regulated by miRNAs such as those analyzed in this study.

It will be necessary to demonstrate the direct relation of these pathways with asthma. In this frame, there is an excellent opportunity to show if these pathways have a relationship with the development of asthma and reverse this disease through their regulation.

Our study has several limitations. The initial sample size of n=3 is very small. The number of control subjects is less than that of the patients; although to correct for this, the groups were matched by age, resulting in a comparison of 27 asthmatic patients versus 27 controls. So, it is necessary to study it in a larger population. In our study, all patients received treatment, so it would be convenient to include subjects in asthmatic crises before treatment to evaluate differences in the expression levels of miRNAs. On the other hand, although the use of multiple endogenous reference genes is considered optimal for normalization of gene expression data, in our study only miR-30e-5p exhibited stable expression across the plasma samples and was therefore selected as the reference control. Additionally, we did not find a correlation between miRNA expression and BMI or FEV1, which could have significant clinical relevance. We also did not analyze the expression of the target genes of these miRNAs, which could provide stronger support for the findings of the *in silico* KEGG analysis. Finally, it will be necessary to replicate the study in a larger population to establish a sensitive and specific AUC (Area Under the Curve) value that can differentiate asthmatics from healthy subjects. Additionally, other respiratory pathologies should be considered to propose these microRNAs as asthma biomarkers. In summary, this study shows that miR-17-5p, miR-18a-5p, miR-106b-5p, and miR-223-3p are downregulated in asthmatic patients compared to healthy controls. To our knowledge, this is the first study to report the dysregulation of these biomolecules in the peripheral blood of patients with asthma. If the distribution in the ROC curves is maintained in future studies, these microRNAs could be considered potential a molecule of preliminary diagnostic value.

## Supplementary Materials

Table S1: patients IgE and Eosinophils. Table S2: Gene union Autophagy; Table S3: Gene union FoxO.

## Figures and Tables

**Table 1:** Demographic and clinical characteristics of the patients included in the study

Variable	HC (n=30)	Asthmatic (n=55)	P value
Sex^a^:			
Women	23 (76.7)	37 (67.1)	0.366
Men	7 (23.3)	18 (32.9)	
Age (years)^b^	41.27 ± 14.23	49.00 ± 13.52	0.009
Weight (kg)^b^	64.10 ± 8.86	69.55 ± 11.94	0.024
BMI (kg/m²)^b^	24.87 ± 2.81	27.96 ± 5.10	<0.001
FEV1 (l)^b^	99.86 ± 14.38	73.66 ± 16.79	<0.001

Data are presented as ^a^ number of subjects (percentage) or as ^b^ mean ± standard deviation. P values were calculated by χ² or Student´s t-test. Abbreviations: HC (Healthy Controls); BMI (Body mass index); FEV1 (Forced expiratory volume during the first second).

**Table 2:** Asthma group, eosinophils count, and IgE levels

Asthmatic group	IgE U/ml	% Eosinophil	Eosinophil count (cells/ml)
	268.1 (SD 474.9)	6.02 (SD 3.78)	451 (SD 340)
*Reference range **Eosinophilic asthma**	>150	>3	>150

*GINA guide

**Table 3:** Fold Change Asthmatic group vs Healthy Control

	Log2 (2^-ΔΔct^ of Asthmatic vs HC)	-Log10 (P-value)
**miR-17-5p**	-22.7	2.1
**miR-18a-5p**	-3.35	2.2
**miR-106b-5p**	-3.61	2.25
**miR-126-3p**	-6.16	2.31
**miR-223-3p**	-30	3.1
**miR-374a-5p**	-21.4	4.71

**Table 4:** Relative levels of circulating miRNAs in plasma from asthmatic patients vs. healthy controls.

	Healthy Controls (n=27)			Asthmatic (n=27)			
		Interquartile Range			Interquartile Range		
	Median	Minimum	Maximum	Median	Minimum	Maximum	p value^a^
miR-17-5p	10.86	0.36	54.55	4.4	1.52	23.99	0.04
miR-18a-5p	0.66	0.04	1.83	0.44	0.012	9.62	0.03
miR-106b-5p	1.42	0.011	303.78	1.12	0.12	2.09	0.023
miR-223-3p	118.58	0.005	2323.00	31.99	14.02	27.58	0.016
