# Supplementary material for: Circulating miRNAs are Down-regulated in Asthmatic Patients; Case-control Study
Source: Int J Med Sci. 2025 Sep 8;22(15):4003–12. doi: 10.7150/ijms.111022 (PMC12492366; doi:10.7150/ijms.111022)
Supplement: Supplementary file 1 — Table S1: patients IgE and Eosinophils. Table S2: Gene union Autophagy; Table S3: Gene union FoxO. [file ijmsv22p4003s1.pdf]

Ashmatic patient.#	EOS%	Eosinophils cells/ml	IGE U/ml
201	4.4	300	6.37
202	8.9	500	149
203	4.7	300	**924**
204	2	100	60.6
209	0.4	0	**630**
210	5.8	500	103
211	8.7	500	42.5
212	2.3	200	104
213	18.3	220	140
214	5.9	500	78.2
215	9.4	600	138
216	3.7	300	114
217	4	400	**215**
218	9.1	700	130
219	4.3	300	149
220	2.6	220	**206**
221	7.6	800	93
222	15.9	1000	22.7
223	1	100	**324**
224	10.1	600	**1600**
225	6.6	400	124
226	6.9	400	26.7
227	6.5	400	76.8
228	4.2	300	145
229	3.1	300	**354**
310	10.1	100	56.5
311	0.7	100	**467**
312	6.6	500	26.4
313	0.5	0	5
315	3.3	300	**636**
316	11.2	900	147
317	6.7	500	7.42
328	1.2	100	**449**
331	4	400	51.1
340	3.2	200	**173**
347	4	200	22.3
348	3.3	200	**200**
351	2.8	200	124
352	4.6	300	73.1
353	8.7	600	25.7
357	7.1	500	73.3
362	11.6	800	24.8
363	4.6	400	90.5
364	6.8	300	22.9
365	12.6	900	**1130**
368	2	100	**204**
370	6.2	500	88.6
376	6.3	500	159
377	6.7	600	**205**
378	4.9	300	**2820**

	380	10.8	700	**480**

FOXO PATHWAY

miRNA IDs	miRNA Names	Target Gene IDs	Target Gene Names
MIMAT0000070	hsa-miR-17-5p	ENSG00000142208	AKT1
MIMAT0000680	hsa-miR-106b-5p	ENSG00000142208	AKT1
MIMAT0000445	hsa-miR-126-3p	ENSG00000105221	AKT2
MIMAT0000070	hsa-miR-17-5p	ENSG00000117020	AKT3
MIMAT0000070	hsa-miR-17-5p	ENSG00000260548	AL035425.2
MIMAT0000070	hsa-miR-17-5p	ENSG00000153094	BCL2L11
MIMAT0000680	hsa-miR-106b-5p	ENSG00000153094	BCL2L11
MIMAT0000070	hsa-miR-17-5p	ENSG00000157764	BRAF
MIMAT0000680	hsa-miR-106b-5p	ENSG00000157764	BRAF
MIMAT0000280	hsa-miR-223-3p	ENSG00000121691	CAT
MIMAT0000070	hsa-miR-17-5p	ENSG00000134057	CCNB1
MIMAT0000680	hsa-miR-106b-5p	ENSG00000134057	CCNB1
MIMAT0000070	hsa-miR-17-5p	ENSG00000110092	CCND1
MIMAT0000680	hsa-miR-106b-5p	ENSG00000110092	CCND1
MIMAT0000070	hsa-miR-17-5p	ENSG00000118971	CCND2
MIMAT0000680	hsa-miR-106b-5p	ENSG00000118971	CCND2
MIMAT0000070	hsa-miR-17-5p	ENSG00000138764	CCNG2
MIMAT0000680	hsa-miR-106b-5p	ENSG00000138764	CCNG2
MIMAT0000070	hsa-miR-17-5p	ENSG00000124762	CDKN1A
MIMAT0000680	hsa-miR-106b-5p	ENSG00000124762	CDKN1A
MIMAT0000070	hsa-miR-17-5p	ENSG00000111276	CDKN1B
MIMAT0000280	hsa-miR-223-3p	ENSG00000213341	CHUK
MIMAT0000070	hsa-miR-17-5p	ENSG00000100393	EP300
MIMAT0000680	hsa-miR-106b-5p	ENSG00000100393	EP300
MIMAT0000070	hsa-miR-17-5p	ENSG00000176165	FOXG1
MIMAT0000070	hsa-miR-17-5p	ENSG00000150907	FOXO1
MIMAT0000280	hsa-miR-223-3p	ENSG00000150907	FOXO1
MIMAT0000280	hsa-miR-223-3p	ENSG00000118689	FOXO3
MIMAT0000445	hsa-miR-126-3p	ENSG00000118689	FOXO3
MIMAT0000070	hsa-miR-17-5p	ENSG00000139112	GABARAPL1
MIMAT0000070	hsa-miR-17-5p	ENSG00000177885	GRB2
MIMAT0000070	hsa-miR-17-5p	ENSG00000140443	IGF1R
MIMAT0000280	hsa-miR-223-3p	ENSG00000140443	IGF1R
MIMAT0000680	hsa-miR-106b-5p	ENSG00000140443	IGF1R
MIMAT0000445	hsa-miR-126-3p	ENSG00000169047	IRS1
MIMAT0000680	hsa-miR-106b-5p	ENSG00000169047	IRS1
MIMAT0000445	hsa-miR-126-3p	ENSG00000185950	IRS2
MIMAT0000680	hsa-miR-106b-5p	ENSG00000185950	IRS2
MIMAT0000070	hsa-miR-17-5p	ENSG00000100030	MAPK1
MIMAT0000680	hsa-miR-106b-5p	ENSG00000100030	MAPK1
MIMAT0000070	hsa-miR-17-5p	ENSG00000156711	MAPK13
MIMAT0000070	hsa-miR-17-5p	ENSG00000107643	MAPK8
MIMAT0000680	hsa-miR-106b-5p	ENSG00000107643	MAPK8
MIMAT0000070	hsa-miR-17-5p	ENSG00000050748	MAPK9
MIMAT0000680	hsa-miR-106b-5p	ENSG00000050748	MAPK9

MIMAT0000070	hsa-miR-17-5p	ENSG00000135679	MDM2
MIMAT0000680	hsa-miR-106b-5p	ENSG00000135679	MDM2
MIMAT0000070	hsa-miR-17-5p	ENSG00000087095	NLK
MIMAT0000680	hsa-miR-106b-5p	ENSG00000087095	NLK
MIMAT0000070	hsa-miR-17-5p	ENSG00000213281	NRAS
MIMAT0000680	hsa-miR-106b-5p	ENSG00000213281	NRAS
MIMAT0000070	hsa-miR-17-5p	ENSG00000140992	PDPK1
MIMAT0000680	hsa-miR-106b-5p	ENSG00000140992	PDPK1
MIMAT0000070	hsa-miR-17-5p	ENSG00000145675	PIK3R1
MIMAT0000680	hsa-miR-106b-5p	ENSG00000145675	PIK3R1
MIMAT0000445	hsa-miR-126-3p	ENSG00000105647	PIK3R2
MIMAT0000070	hsa-miR-17-5p	ENSG00000117461	PIK3R3
MIMAT0000070	hsa-miR-17-5p	ENSG00000166851	PLK1
MIMAT0000680	hsa-miR-106b-5p	ENSG00000166851	PLK1
MIMAT0000445	hsa-miR-126-3p	ENSG00000145632	PLK2
MIMAT0000680	hsa-miR-106b-5p	ENSG00000132356	PRKAA1
MIMAT0000070	hsa-miR-17-5p	ENSG00000162409	PRKAA2
MIMAT0000070	hsa-miR-17-5p	ENSG00000131791	PRKAB2
MIMAT0000680	hsa-miR-106b-5p	ENSG00000131791	PRKAB2
MIMAT0000070	hsa-miR-17-5p	ENSG00000181929	PRKAG1
MIMAT0000070	hsa-miR-17-5p	ENSG00000171862	PTEN
MIMAT0000680	hsa-miR-106b-5p	ENSG00000171862	PTEN
MIMAT0000070	hsa-miR-17-5p	ENSG00000103479	RBL2
MIMAT0000680	hsa-miR-106b-5p	ENSG00000103479	RBL2
MIMAT0000070	hsa-miR-17-5p	ENSG00000170989	S1PR1
MIMAT0000680	hsa-miR-106b-5p	ENSG00000170989	S1PR1
MIMAT0000070	hsa-miR-17-5p	ENSG00000145391	SETD7
MIMAT0000445	hsa-miR-126-3p	ENSG00000118515	SGK1
MIMAT0000070	hsa-miR-17-5p	ENSG00000141646	SMAD4
MIMAT0000680	hsa-miR-106b-5p	ENSG00000141646	SMAD4
MIMAT0000070	hsa-miR-17-5p	ENSG00000112096	SOD2
MIMAT0000680	hsa-miR-106b-5p	ENSG00000112096	SOD2
MIMAT0000680	hsa-miR-106b-5p	ENSG00000115904	SOS1
MIMAT0000070	hsa-miR-17-5p	ENSG00000100485	SOS2
MIMAT0000070	hsa-miR-17-5p	ENSG00000168610	STAT3
MIMAT0000280	hsa-miR-223-3p	ENSG00000168610	STAT3
MIMAT0000680	hsa-miR-106b-5p	ENSG00000168610	STAT3
MIMAT0000070	hsa-miR-17-5p	ENSG00000118046	STK11
MIMAT0000445	hsa-miR-126-3p	ENSG00000118046	STK11
MIMAT0000680	hsa-miR-106b-5p	ENSG00000118046	STK11
MIMAT0000680	hsa-miR-106b-5p	ENSG00000106799	TGFBR1
MIMAT0000070	hsa-miR-17-5p	ENSG00000163513	TGFBR2
MIMAT0000680	hsa-miR-106b-5p	ENSG00000163513	TGFBR2
MIMAT0000070	hsa-miR-17-5p	ENSG00000187555	USP7

**Targets Resource**
TarBase v8.0
TarBase v8.0
TarBase v8.0
TarBase v8.0
TarBase v8.0
TarBase v8.0
TarBase v8.0
TarBase v8.0
TarBase v8.0
TarBase v8.0
TarBase v8.0
TarBase v8.0
TarBase v8.0
TarBase v8.0
TarBase v8.0
TarBase v8.0
TarBase v8.0
TarBase v8.0
TarBase v8.0
TarBase v8.0
TarBase v8.0
TarBase v8.0
TarBase v8.0
TarBase v8.0
TarBase v8.0
TarBase v8.0
TarBase v8.0
TarBase v8.0
TarBase v8.0
TarBase v8.0
TarBase v8.0
TarBase v8.0
TarBase v8.0
TarBase v8.0
TarBase v8.0
TarBase v8.0
TarBase v8.0
TarBase v8.0
TarBase v8.0
TarBase v8.0
TarBase v8.0
TarBase v8.0
TarBase v8.0
TarBase v8.0
TarBase v8.0

TarBase v8.0
TarBase v8.0
TarBase v8.0
TarBase v8.0
TarBase v8.0
TarBase v8.0
TarBase v8.0
TarBase v8.0
TarBase v8.0
TarBase v8.0
TarBase v8.0
TarBase v8.0
TarBase v8.0
TarBase v8.0
TarBase v8.0
TarBase v8.0
TarBase v8.0
TarBase v8.0
TarBase v8.0
TarBase v8.0
TarBase v8.0
TarBase v8.0
TarBase v8.0
TarBase v8.0
TarBase v8.0
TarBase v8.0
TarBase v8.0
TarBase v8.0
TarBase v8.0
TarBase v8.0
TarBase v8.0
TarBase v8.0
TarBase v8.0
TarBase v8.0
TarBase v8.0
TarBase v8.0
TarBase v8.0
TarBase v8.0
TarBase v8.0
TarBase v8.0
TarBase v8.0
TarBase v8.0
TarBase v8.0
TarBase v8.0

AUTOPHAGY PATHWAY

miRNA IDs	miRNA Names	Target Gene IDs	Target Gene Names
MIMAT0000680	hsa-miR-106b-5p	ENSG00000025039	RRAGD
MIMAT0000680	hsa-miR-106b-5p	ENSG00000050748	MAPK9
MIMAT0000680	hsa-miR-106b-5p	ENSG00000066739	ATG2B
MIMAT0000680	hsa-miR-106b-5p	ENSG00000078142	PIK3C3
MIMAT0000680	hsa-miR-106b-5p	ENSG00000078804	TP53INP2
MIMAT0000680	hsa-miR-106b-5p	ENSG00000085978	ATG16L1
MIMAT0000680	hsa-miR-106b-5p	ENSG00000097033	SH3GLB1
MIMAT0000680	hsa-miR-106b-5p	ENSG00000100030	MAPK1
MIMAT0000680	hsa-miR-106b-5p	ENSG00000107643	MAPK8
MIMAT0000680	hsa-miR-106b-5p	ENSG00000113575	PPP2CA
MIMAT0000680	hsa-miR-106b-5p	ENSG00000118046	STK11
MIMAT0000680	hsa-miR-106b-5p	ENSG00000132356	PRKAA1
MIMAT0000680	hsa-miR-106b-5p	ENSG00000133818	RRAS2
MIMAT0000680	hsa-miR-106b-5p	ENSG00000134001	EIF2S1
MIMAT0000680	hsa-miR-106b-5p	ENSG00000140443	IGF1R
MIMAT0000680	hsa-miR-106b-5p	ENSG00000140992	PDPK1
MIMAT0000680	hsa-miR-106b-5p	ENSG00000142208	AKT1
MIMAT0000680	hsa-miR-106b-5p	ENSG00000142875	PRKACB
MIMAT0000680	hsa-miR-106b-5p	ENSG00000144848	ATG3
MIMAT0000680	hsa-miR-106b-5p	ENSG00000145016	RUBCN
MIMAT0000680	hsa-miR-106b-5p	ENSG00000145675	PIK3R1
MIMAT0000680	hsa-miR-106b-5p	ENSG00000148572	NRBF2
MIMAT0000680	hsa-miR-106b-5p	ENSG00000161011	SQSTM1
MIMAT0000680	hsa-miR-106b-5p	ENSG00000165861	ZFYVE1
MIMAT0000680	hsa-miR-106b-5p	ENSG00000169047	IRS1
MIMAT0000680	hsa-miR-106b-5p	ENSG00000171862	PTEN
MIMAT0000680	hsa-miR-106b-5p	ENSG00000176994	SMCR8
MIMAT0000680	hsa-miR-106b-5p	ENSG00000177169	ULK1
MIMAT0000680	hsa-miR-106b-5p	ENSG00000185950	IRS2
MIMAT0000680	hsa-miR-106b-5p	ENSG00000213281	NRAS
MIMAT0000445	hsa-miR-126-3p	ENSG00000105221	AKT2
MIMAT0000445	hsa-miR-126-3p	ENSG00000105647	PIK3R2
MIMAT0000445	hsa-miR-126-3p	ENSG00000118046	STK11
MIMAT0000445	hsa-miR-126-3p	ENSG00000161011	SQSTM1
MIMAT0000445	hsa-miR-126-3p	ENSG00000169047	IRS1
MIMAT0000445	hsa-miR-126-3p	ENSG00000171791	BCL2
MIMAT0000445	hsa-miR-126-3p	ENSG00000185950	IRS2
MIMAT0000070	hsa-miR-17-5p	ENSG00000025039	RRAGD
MIMAT0000070	hsa-miR-17-5p	ENSG00000050748	MAPK9
MIMAT0000070	hsa-miR-17-5p	ENSG00000066739	ATG2B
MIMAT0000070	hsa-miR-17-5p	ENSG00000078142	PIK3C3
MIMAT0000070	hsa-miR-17-5p	ENSG00000085978	ATG16L1
MIMAT0000070	hsa-miR-17-5p	ENSG00000089289	IGBP1
MIMAT0000070	hsa-miR-17-5p	ENSG00000099940	SNAP29
MIMAT0000070	hsa-miR-17-5p	ENSG00000100030	MAPK1

MIMAT0000070	hsa-miR-17-5p	ENSG00000100330	MTMR3
MIMAT0000070	hsa-miR-17-5p	ENSG00000100644	HIF1A
MIMAT0000070	hsa-miR-17-5p	ENSG00000107643	MAPK8
MIMAT0000070	hsa-miR-17-5p	ENSG00000108389	MTMR4
MIMAT0000070	hsa-miR-17-5p	ENSG00000110497	AMBRA1
MIMAT0000070	hsa-miR-17-5p	ENSG00000113575	PPP2CA
MIMAT0000070	hsa-miR-17-5p	ENSG00000117020	AKT3
MIMAT0000070	hsa-miR-17-5p	ENSG00000117461	PIK3R3
MIMAT0000070	hsa-miR-17-5p	ENSG00000118046	STK11
MIMAT0000070	hsa-miR-17-5p	ENSG00000134001	EIF2S1
MIMAT0000070	hsa-miR-17-5p	ENSG00000138069	RAB1A
MIMAT0000070	hsa-miR-17-5p	ENSG00000139112	GABARAPL1
MIMAT0000070	hsa-miR-17-5p	ENSG00000140443	IGF1R
MIMAT0000070	hsa-miR-17-5p	ENSG00000140992	PDPK1
MIMAT0000070	hsa-miR-17-5p	ENSG00000142208	AKT1
MIMAT0000070	hsa-miR-17-5p	ENSG00000142875	PRKACB
MIMAT0000070	hsa-miR-17-5p	ENSG00000144848	ATG3
MIMAT0000070	hsa-miR-17-5p	ENSG00000145016	RUBCN
MIMAT0000070	hsa-miR-17-5p	ENSG00000145675	PIK3R1
MIMAT0000070	hsa-miR-17-5p	ENSG00000148572	NRBF2
MIMAT0000070	hsa-miR-17-5p	ENSG00000157954	WIPI2
MIMAT0000070	hsa-miR-17-5p	ENSG00000161011	SQSTM1
MIMAT0000070	hsa-miR-17-5p	ENSG00000162409	PRKAA2
MIMAT0000070	hsa-miR-17-5p	ENSG00000167657	DAPK3
MIMAT0000070	hsa-miR-17-5p	ENSG00000171552	BCL2L1
MIMAT0000070	hsa-miR-17-5p	ENSG00000171791	BCL2
MIMAT0000070	hsa-miR-17-5p	ENSG00000171862	PTEN
MIMAT0000070	hsa-miR-17-5p	ENSG00000177169	ULK1
MIMAT0000070	hsa-miR-17-5p	ENSG00000189403	HMGB1
MIMAT0000070	hsa-miR-17-5p	ENSG00000196730	DAPK1
MIMAT0000070	hsa-miR-17-5p	ENSG00000198793	MTOR
MIMAT0000070	hsa-miR-17-5p	ENSG00000198925	ATG9A
MIMAT0000070	hsa-miR-17-5p	ENSG00000213281	NRAS
MIMAT0000070	hsa-miR-17-5p	ENSG00000260548	AL035425.2
MIMAT0000280	hsa-miR-223-3p	ENSG00000140443	IGF1R
MIMAT0000280	hsa-miR-223-3p	ENSG00000168209	DDIT4
MIMAT0000680	hsa-miR-106b-5p	ENSG00000168610	STAT3
MIMAT0000070	hsa-miR-17-5p	ENSG00000118046	STK11
MIMAT0000445	hsa-miR-126-3p	ENSG00000118046	STK11
MIMAT0000680	hsa-miR-106b-5p	ENSG00000118046	STK11
MIMAT0000680	hsa-miR-106b-5p	ENSG00000106799	TGFBR1
MIMAT0000070	hsa-miR-17-5p	ENSG00000163513	TGFBR2
MIMAT0000680	hsa-miR-106b-5p	ENSG00000163513	TGFBR2
MIMAT0000070	hsa-miR-17-5p	ENSG00000187555	USP7

Targets Resource
TarBase v8.0
TarBase v8.0
TarBase v8.0
TarBase v8.0
TarBase v8.0
TarBase v8.0
TarBase v8.0
TarBase v8.0
TarBase v8.0
TarBase v8.0
TarBase v8.0
TarBase v8.0
TarBase v8.0
TarBase v8.0
TarBase v8.0
TarBase v8.0
TarBase v8.0
TarBase v8.0
TarBase v8.0
TarBase v8.0
TarBase v8.0
TarBase v8.0
TarBase v8.0
TarBase v8.0
TarBase v8.0
TarBase v8.0
TarBase v8.0
TarBase v8.0
TarBase v8.0
TarBase v8.0
TarBase v8.0
TarBase v8.0
TarBase v8.0
TarBase v8.0
TarBase v8.0
TarBase v8.0
TarBase v8.0
TarBase v8.0
TarBase v8.0
TarBase v8.0
TarBase v8.0
TarBase v8.0
TarBase v8.0
TarBase v8.0
TarBase v8.0

TarBase v8.0
TarBase v8.0
TarBase v8.0
TarBase v8.0
TarBase v8.0
TarBase v8.0
TarBase v8.0
TarBase v8.0
TarBase v8.0
TarBase v8.0
TarBase v8.0
TarBase v8.0
TarBase v8.0
TarBase v8.0
TarBase v8.0
TarBase v8.0
TarBase v8.0
TarBase v8.0
TarBase v8.0
TarBase v8.0
TarBase v8.0
TarBase v8.0
TarBase v8.0
TarBase v8.0
TarBase v8.0
TarBase v8.0
TarBase v8.0
TarBase v8.0
TarBase v8.0
TarBase v8.0
TarBase v8.0
TarBase v8.0
TarBase v8.0
TarBase v8.0
TarBase v8.0
TarBase v8.0
TarBase v8.0
TarBase v8.0
TarBase v8.0
TarBase v8.0
TarBase v8.0
TarBase v8.0
TarBase v8.0
TarBase v8.0
